# Supplementary material for: Biologically derived epicardial patch induces macrophage mediated pathophysiologic repair in chronically infarcted swine hearts
Source: Commun Biol. 2023 Nov 25;6:1203. doi: 10.1038/s42003-023-05564-w (PMC10676365; doi:10.1038/s42003-023-05564-w)
Supplement: Supplementary file 1 — Reporting Summary [file 42003_2023_5564_MOESM1_ESM.pdf]

nature portfolio

Corresponding author(s): Steven Goldman MD

Last updated by author(s): Sep 29, 2023

Reporting Summary

Nature Portfolio wishes to improve the reproducibility of the work that we publish. This form provides structure for consistency and transparency in reporting. For further information on Nature Portfolio policies, see our Editorial Policies and the Editorial Policy Checklist.

Statistics

For all statistical analyses, confirm that the following items are present in the figure legend, table legend, main text, or Methods section.

n/a	Confirmed	
☐	☒	The exact sample size (*n*) for each experimental group/condition, given as a discrete number and unit of measurement
☐	☒	A statement on whether measurements were taken from distinct samples or whether the same sample was measured repeatedly
☐	☒	The statistical test(s) used AND whether they are one- or two-sided<br>*Only common tests should be described solely by name; describe more complex techniques in the Methods section.*
☐	☒	A description of all covariates tested
☐	☒	A description of any assumptions or corrections, such as tests of normality and adjustment for multiple comparisons
☐	☒	A full description of the statistical parameters including central tendency (e.g. means) or other basic estimates (e.g. regression coefficient) AND variation (e.g. standard deviation) or associated estimates of uncertainty (e.g. confidence intervals)
☐	☒	For null hypothesis testing, the test statistic (e.g. *F*, *t*, *r*) with confidence intervals, effect sizes, degrees of freedom and *P* value noted<br>*Give P values as exact values whenever suitable.*
☒	☐	For Bayesian analysis, information on the choice of priors and Markov chain Monte Carlo settings
☒	☐	For hierarchical and complex designs, identification of the appropriate level for tests and full reporting of outcomes
☒	☐	Estimates of effect sizes (e.g. Cohen's *d*, Pearson's *r*), indicating how they were calculated

Our web collection on statistics for biologists contains articles on many of the points above.

Software and code

Policy information about availability of computer code

Data collection	For hemodynamic and PV analysis we utilized ADInstrument's LabChart8 (version 8.1.25). For GeoMX DSP collection we used version v2.1 of the GeoMX DSP software.
Data analysis	Data analysis was completed with SigmaPlot (version 12.5), RStudio (version 4.1.2),. Packages used are GeomxTools (Version 3.2.0, 33) and NanostringNCTools (Version 1.6.0, 34)). Spatial deconvolution was achieved with SpatialDecon (Version 1.8.0, 35).

For manuscripts utilizing custom algorithms or software that are central to the research but not yet described in published literature, software must be made available to editors and reviewers. We strongly encourage code deposition in a community repository (e.g. GitHub). See the Nature Portfolio guidelines for submitting code & software for further information.

Data

Policy information about availability of data

All manuscripts must include a data availability statement. This statement should provide the following information, where applicable:

- Accession codes, unique identifiers, or web links for publicly available datasets
- A description of any restrictions on data availability
- For clinical datasets or third party data, please ensure that the statement adheres to our policy

The datasets and computational code used and/or analyzed during the current study are available from the corresponding author on reasonable request.

Research involving human participants, their data, or biological material

Policy information about studies with human participants or human data. See also policy information about sex, gender (identity/presentation), and sexual orientation and race, ethnicity and racism.

Reporting on sex and gender	N/A
Reporting on race, ethnicity, or other socially relevant groupings	N/A
Population characteristics	N/A
Recruitment	N/A
Ethics oversight	N/A

Note that full information on the approval of the study protocol must also be provided in the manuscript.

Field-specific reporting

Please select the one below that is the best fit for your research. If you are not sure, read the appropriate sections before making your selection.

☒ Life sciences ☐ Behavioural & social sciences ☐ Ecological, evolutionary & environmental sciences

For a reference copy of the document with all sections, see nature.com/documents/nr-reporting-summary-flat.pdf

Life sciences study design

All studies must disclose on these points even when the disclosure is negative.

Sample size	The number of animals for each objective is designed for N=10 for each data point. Current data collection and data analysis shows that N=10 will provide 80% power to show a 20% difference in MRI and hemodynamic related endpoints. A normal group of N=4 would be used to reduce the total number of pigs needed for this aim.
Data exclusions	Two swine were excluded from CMR data due to heart rates >30 beats/min than baseline rates.
Replication	All attempts were made for data reproducibility.
Randomization	All studies were randomized.
Blinding	Operators that performed the studies were blinded to the treatment protocols.

Reporting for specific materials, systems and methods

We require information from authors about some types of materials, experimental systems and methods used in many studies. Here, indicate whether each material, system or method listed is relevant to your study. If you are not sure if a list item applies to your research, read the appropriate section before selecting a response.

Materials & experimental systems

n/a	Involved in the study	
☐	☒	Antibodies
☐	☒	Eukaryotic cell lines
☒	☐	Palaeontology and archaeology
☐	☒	Animals and other organisms
☒	☐	Clinical data
☒	☐	Dual use research of concern
☒	☐	Plants

Methods

n/a	Involved in the study	
☒	☐	ChIP-seq
☒	☐	Flow cytometry
☒	☐	MRI-based neuroimaging

Antibodies

Antibodies used	In this study we utilized CD45 (Rat monoclonal (30-F11) Alexa Fluor® 647), desmin (Desmin (RD301) Alexa Fluor® 594), SYTO 13, and α-SMA (ab184675 Alexa Fluor® 488)

Validation	Antibodies were pre-validated by NanoString and openly available

Eukaryotic cell lines

Policy information about cell lines and Sex and Gender in Research

Cell line source(s)	A proprietary bioresorbable mesh was cut to 5cm diameter disks, sterilized using ethylene oxide and allowed to degas for two weeks. Previously expanded NDFs and hiPSC-CMs were thawed and cultured to incorporate them into the sterile matrix disks. Cultures were maintained at 5°C and 5% CO2 for 30 days. We used hiPSC-CMs that express more than 90% cardiac troponin T CMs. The human NDFs .were obtained from human foreskin and were expanded for patch fabrication. The cardiomyocytes undergo a 20-day differentiation process, patches contain a cardiomyocyte-to-fibroblast ratio of 1:1 to 2:1.
Authentication	We used hiPSC-CMs that express more than 90% cardiac troponin T CMs. The human NDFs were obtained from human foreskin and were expanded for patch fabrication.
Mycoplasma contamination	N/A
Commonly misidentified lines (See ICLAC register)	N/A

Animals and other research organisms

Policy information about studies involving animals; ARRIVE guidelines recommended for reporting animal research, and Sex and Gender in Research

Laboratory animals	Yucatan miniature swine (male), C57BL/6J mice (male)
Wild animals	N/A
Reporting on sex	Analysis was completed in males.
Field-collected samples	N/A
Ethics oversight	All animal work was performed with oversight of the University of Arizona Institutional Animal Care and Use Committee (IACUC) and the University of Arizona Animal Care (UAC) veterinary staff. The IACUC oversees the University of Arizona's animal care and use program and is responsible for reviewing and approving all activities utilizing vertebrate animals for research, teaching, and testing. Compliance Information: USDA, Class R Research Facility, Registration Number: 86-R-0003, Expiration Date: August 24, 2022. NIH/OLAW Assurance Number: D16-00159, Expiration Date: August 31, 2023 and AAALAC International Accredited since 1969, Accreditation Number: 000163, Status: Continued Full Accreditation.

Note that full information on the approval of the study protocol must also be provided in the manuscript.
